# Widespread of horizontal gene transfer in the human genome

**DOI:** 10.1186/s12864-017-3649-y

**Published:** 2017-04-04

**Authors:** Wenze Huang, Lillian Tsai, Yulong Li, Nan Hua, Chen Sun, Chaochun Wei

**Affiliations:** 1 School of Life Sciences and Biotechnology, Shanghai Jiao Tong University, 800 Dongchuan Road, Shanghai, 200240 China; 2 Harvard College, Harvard University, Cambridge, 02138 MA USA; 3 Shanghai Center for Bioinformation Technology, 1278 Keyuan Road, Pudong District, Shanghai, 201203 China

## Abstract

**Background:**

A fundamental concept in biology is that heritable material is passed from parents to offspring, a process called vertical gene transfer. An alternative mechanism of gene acquisition is through horizontal gene transfer (HGT), which involves movement of genetic materials between different species. Horizontal gene transfer has been found prevalent in prokaryotes but very rare in eukaryote. In this paper, we investigate horizontal gene transfer in the human genome.

**Results:**

From the pair-wise alignments between human genome and 53 vertebrate genomes, 1,467 human genome regions (2.6 M bases) from all chromosomes were found to be more conserved with non-mammals than with most mammals. These human genome regions involve 642 known genes, which are enriched with ion binding. Compared to known horizontal gene transfer regions in the human genome, there were few overlapping regions, which indicated horizontal gene transfer is more common than we expected in the human genome.

**Conclusions:**

Horizontal gene transfer impacts hundreds of human genes and this study provided insight into potential mechanisms of HGT in the human genome.

## Background

The acquisition of genes from an organism other than a direct ancestor, which is called horizontal gene transfer (HGT), is well known in bacteria and unicellular eukaryotes [1–6]. Although the transfer of genes is thought to be crucial in prokaryotic evolution, its existence in higher organisms, including animals, is less well established [7–9]. However, in recent years, more and more instances of horizontal gene transfer have been reported in multicellular eukaryotes, even in humans [7–12]. This phenomenon has been previously reported in bovine genome, which has a high abundance of the BovB sequence of the python and copperhead reptiles. However, the BovB sequence was more similar between cows and reptiles than cows and horses. A proposed mechanism for this phenomenon was horizontal gene transfer between organisms with ticks as the media [13]. Another project discovered the “Space Invaders,” or SPIN elements, that were highly conserved between seven distantly related species from diverse lineages. A proposed mechanism to explain the similarity of sequences between two species with a very distant common ancestor was horizontal gene transfer [9].

A recent study investigated the possibility of HGT in 26 animal species (10 primates, 12 flies and four nematodes) and a simplified analysis in a further 14 vertebrates. Genome-wide comparative and phylogenetic analyses show that HGT in animals typically gives rise to tens or hundreds of active ‘foreign’ genes, largely concerned with metabolism. This analyses suggest that while fruit flies and nematodes have continued to acquire foreign genes throughout their evolution, humans and other primates have gained relatively few since their common ancestor [10].

In this study, we analyzed the pair-wise alignment data between the human reference genome hg19 [14] and 53 other vertebrates (41 mammalian vertebrates and 12 non-mammalian vertebrates). We identified potential HGT regions in the human genome (see Methods). Genome annotations and genome-wide signals from recent genomics or epigenetics studies [15, 16] of these sequences have been gathered to infer the functions of these predicted HGT regions, and evolutionary trends of these sequences have been analyzed. This study thus sheds light upon the prevalence of HGT and provide an insight of its underlining mechanisms.

## Methods

### Pair-wise alignments between the human genome and 53 vertebrate genomes

We started with the pair-wise blastz alignments between the human reference genome hg19 [14] and 53 other vertebrates (41 mammalian vertebrates and 12 non-mammalian vertebrates). The alignment data were then linked into chains and formed into an alignment net [17]. More details about the pair-wise alignment data can be found in the UCSC Genome Browser website [18].

### HGT identification pipeline

**Fig. 1:**
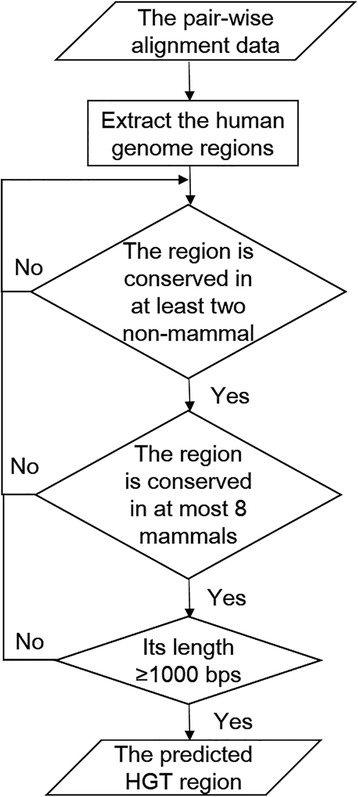
HGT identification pipeline. From the pair-wise alignment data, we extracted the human genome regions that were conserved between 12 non-mammalian vertebrates and the human genome. A human genome region is called a non-mammal conserved region if this region is aligned to at least two non-mammal genomes with an identity no less than 40%. These regions were then compared to the mammalian genomes. For these non-mammal conserved regions, if a region can be aligned to at most eight (<20%) of the 41 mammals with an alignment identity ≥40% and length of the aligned region longer than 40% of the human region, this region is defined as a predicted HGT region. We choose the predicted HGT regions which lengths are longer than 1000 bps to analyze

From the pair-wise alignment data, we identified the human genome regions that were conserved between 12 non-mammalian vertebrates and the human genome. These regions were then compared to the mammalian genomes to create HGT regions. A human genome region is called a non-mammal conserved region if this region is aligned to at least two non-mammal genomes with an identity no less than 40% and the aligned human genome region length is longer than 1000 bps. For these non-mammal conserved regions, if a region can be aligned to at most 8 (<20%) of the 41 mammals with an alignment identity ≥40% and length of the aligned region longer than 40% of the human region, this region is defined as a predicted HGT region (Fig. 1).

It’s notable that this set of predicted HGT regions are not comprehensive since some regions may be separated by small gaps between alignment sequences that cause two short, yet close, conserved sequences to be filtered out. In order to check the reliability of HGT predictions, we explored various identity thresholds in the pipeline (40%, 50 and 60%) between vertebrates and the human genome and the length coverage (40%, 20% and 0) between mammals and the human genome to calculate the number of predicted or candidate HGT regions.

### Conservation analysis of predicted HGT regions

To verify the conservation levels of the sequences we extracted, we randomly sampled 50 sequences from our results and verified the sequences based on the existence of homologous sequence in several groups of vertebrates using the University of California Santa Cruz (UCSC) Comparative Genomics track (Primate Chain/Net, Placental Chain/Net, Vertebrate Chain/Net) [17, 19, 20]. We grouped the 53 vertebrates into two bins: the first bin includes all non-human mammals; the second bin includes all non-mammal vertebrates. For each of our samples, we determined if less than eight mammals have homology sequences with ≥40% identity with hg19 and ≥ 40% of the human genome region length covered.

### Genes Overlapping predicted HGT regions

We checked whether our sets of predicted HGT sequences overlapped with known coding genes in ENSEMBL database [21]. We downloaded the *Homo sapiens* gene data from ENSEMBL database and converted them into bed format file. For each sequences dataset, we intersected the Ensembl Genes with these sequences to discover genes that had any overlap with the predicted HGT regions.

### Coding status analysis

We extracted the transcript IDs from the overlapped Ensembl genes and searched these IDs through the Ensembl Human Gene sets table, in which these genes were classified as protein coding, pseudogenes, etc. [21].

### Gene enrichment analysis

All involved genes were generated from Ensemble Genes [21] and enrichment of these genes was calculated using the Database for Annotation, Visualization and Integrated Discovery (DAVID [22, 23], version 6.7) with all human genes as the background level. All items were adjusted with the modified Fisher Exact p-value (EASE score) < 0.05. The gene enrichment results were shown in three aspects: 1) molecular functions, 2) biological processes, and 3) cellular components.

### Chromatin state analysis

Using the Broad chromHMM [15] Chromatin State track in nine cell lines (Gm12878, H1hesc, Hepg2, Hmec, Hsmm, Huvec, K562, Nhek, Nhlf), we checked the chromatin states of HGT regions. We then compared the percentages of different chromatin states in these regions against the corresponding values in the whole human genome as the background.

### Repeat analysis

We used the RepeatMasker track from the UCSC Genome Browser to generate distributions of the different types of repeats, including SINEs, LINEs, LTRs, DNA repeats and simple repeats [18]. We divided the HGT regions and flanking regions into five regions: w1 (300 bps upstream regions of the HGT regions), w2 (the beginning 300 bps of the HGT regions), w3 (the middle regions of the HGT regions), w4 (the ending 300 bps of the HGT regions), and w5 (300 bps downstream of the HGT regions). We calculated the average proportions of different types of repeats in the five regions of our sequences and drew the average proportions curve of different types of repeats within the HGT regions and 300 bps upstream and downstream of the HGT regions.

### GC content analysis

We calculated the GC content and drew GC content curve for the HGT regions with a similar approach as the above section we did for repeats.

### Histone modification analysis

In order to predict regulatory functions of our sequence, regulatory related histone modification and histone variants on the sequences in H1-hESC cell lines was investigated. One transcription related histone marks: H3K4Me1; and four regulation-related histone marks and variations: H2A.Z, H3K4Me3, H3K9Ac, and H3K27Ac [24] were applied. In addition, one histone methylation related to polycomb-repression: H3K27Me3 [16, 24] was also included. All these datasets were downloaded from the corresponding tracks in the UCSC genome browser.

### Highlighting conservation in phylogenetic tree

We took the phylogenetic tree directly from the Comparative Genomics track “Vertebrate Multiz Alignment & Conservation (100 Species)” [25, 26]. For each HGT region, we extracted the homologous sequences from related vertebrate genomes. Then we aligned these homology sequences by ClustalW and constructed the phylogenetic tree by Maximum Likelihood Method using MEGA6 [27]. We compared this HGT homologous sequence based phylogenetic tree with the species phylogenetic tree and analyzed the differences between two phylogenetic trees. In order to show the predicted HGT events between these species, we used Sprit [28] to calculate the minimum subtree prune and regraft (SPR) distance between the species phylogenetic tree and the HGT homology sequence based phylogenetic tree.

### Searching the homologous sequences in media species

In order to check the confidence levels of the HGT regions found in the human genome, we checked the possible transfer paths from other species to human. 22 HGT fragments (similarity between vertebrates and hg19 was larger than 60% with lengths at least 1000 bps and no alignment between mammalian genomes and hg19 was allowed) were aligned with blastn to the Nucleotide collection database of National Center for Biotechnology Information (NCBI), which consists of all nucleotide sequences available. We searched the Nucleotide collection database to find the homologous sequences of our HGT fragments in some species, which might be the media species for the HGT progress, such as parasites, bacteria or fungi.

## Results

In our pipeline (see Methods), we compared pair-wise alignment block regions between 53 vertebrates and the human genome and extracted those human genome sequences that show greater conservation with non-mammalian vertebrates than with most mammals. With the similarity threshold (40%) and the length coverage (40%) between mammalian genomes and hg19, our pipeline has found 1,467 HGT regions longer than 1,000 bps.

### The location bias of predicted HGT regions in chromosomes

**Fig. 2:**
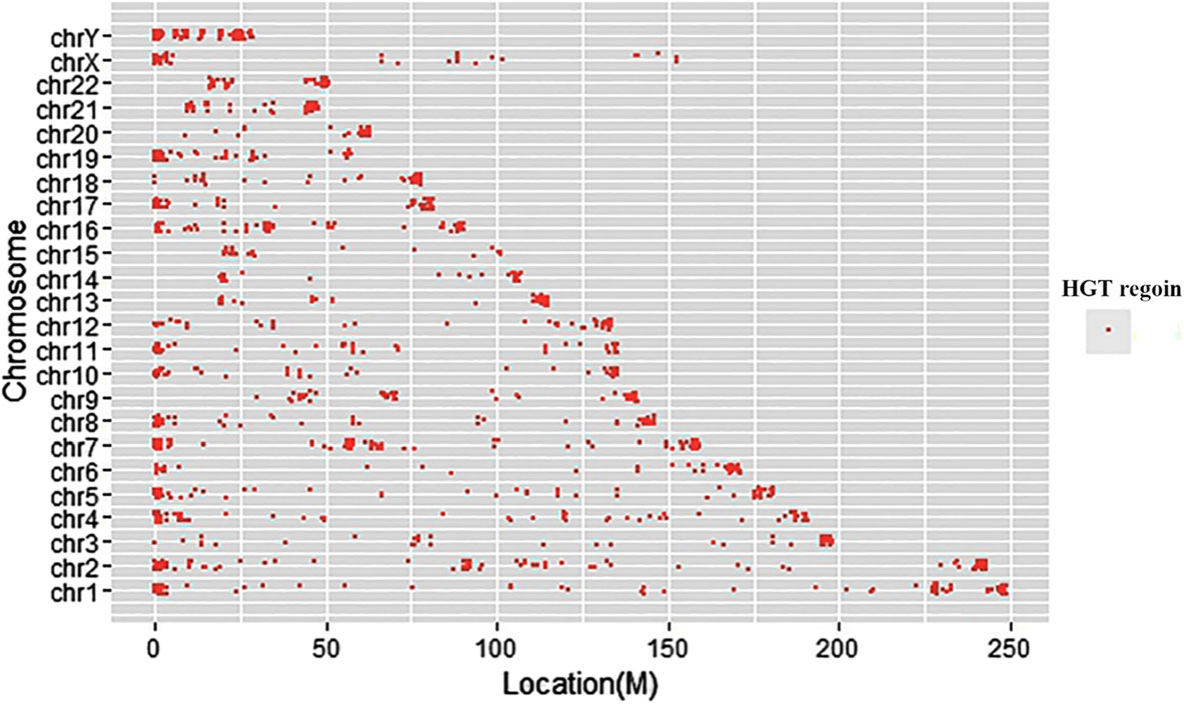
Distribution of predicted HGT regions in chromosomes of the human genome. The location of HGT regions in each chromosome was shown. The thresholds used in the identification pipeline were identity 40%, aligned human genome region length longer than 1000 bps, and the coverage higher than 40% of the human genome regions. The total number of HGT regions was 1,467

We located these HGT regions on the human chromosomes. The number of HGT regions varies from chromosome to chromosome and most of HGT regions are located on both ends of the chromosomes (Table 1, Fig. 2). We calculated the frequency of HGT regions in the both ends and the middle of the chromosomes, and found the frequency of HGT regions in the both ends of the chromosomes is significantly greater than the middle of the chromosomes (paired *t*-test *p*-value < 0.001).

**Table 1:** Distribution of predicted HGT regions in chromosomes of the human genome

Chromosome	Number	Chromosome	Number	Chromosome	Number
chr1	83	chr9	56	chr17	50
chr2	101	chr10	70	chr18	51
chr3	43	chr11	49	chr19	62
chr4	76	chr12	65	chr20	37
chr5	76	chr13	50	chr21	43
chr6	42	chr14	33	chr22	45
chr7	118	chr15	20	chrX	54
chr8	64	chr16	72	chrY	107

Since the HGT regions we predicted here happened after the split of primates from mammals, this discovery leads us to conjecture that the HGT fragments might be more likely to insert on both ends of the chromosomes.

### Analysis of overlapped genes

**Fig. 3:**
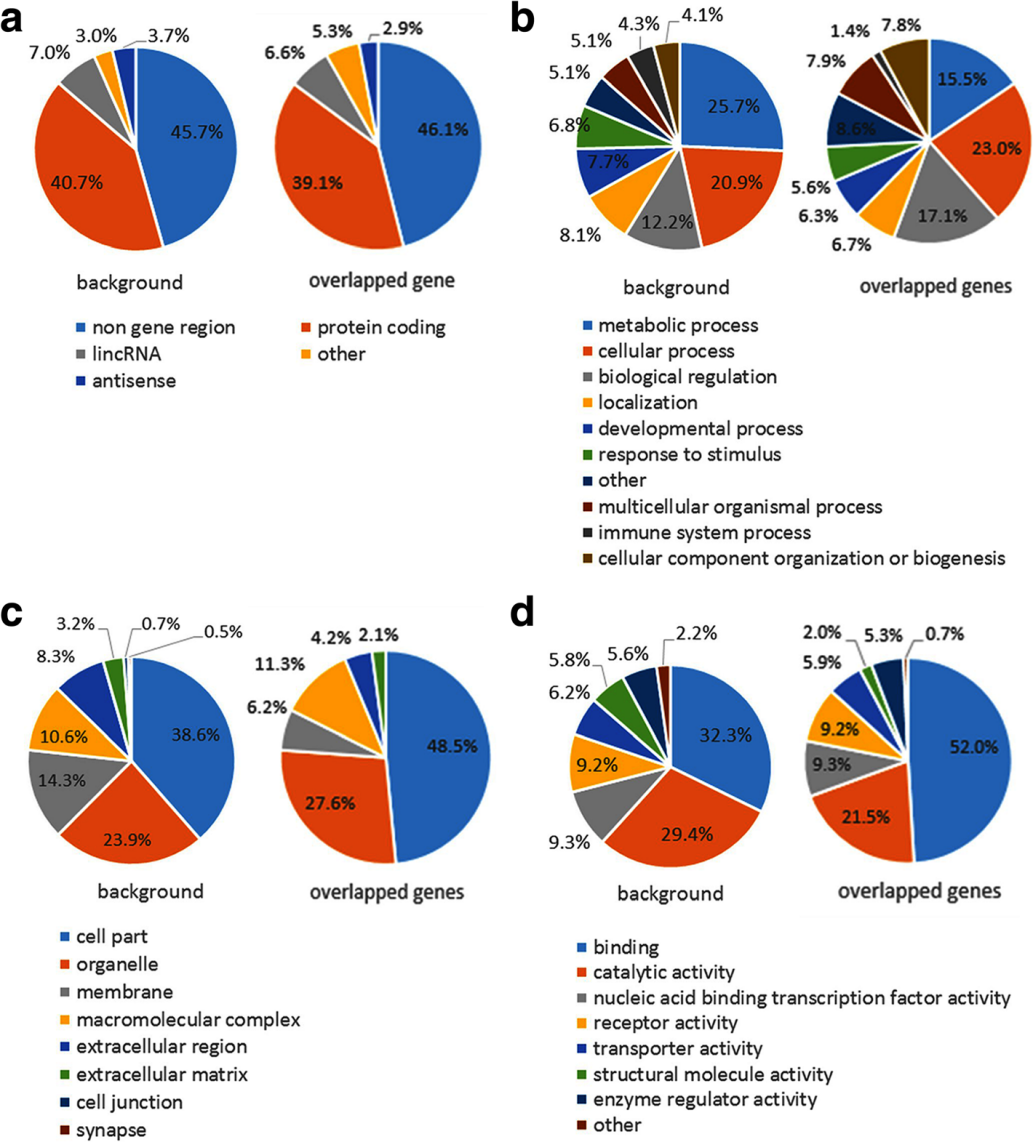
**a** The types of genes overlapping HGT regions. The overlapped Ensembl genes were extracted and Ensembl Source table were searched. The overlapped genes were identified as protein coding, lincRNA, antisense and other (processed transcript, processed pseudogene, etc.). Our pie chart compared the gene type proportions of overlapped genes against whole gene of human as the background. **b**, **c** and **d** Gene enrichment analysis. The gene enrichment results were shown in three aspects, biological processes (Fig. 2b), cellular components (Fig. 2c), and molecular functions (Fig. 2d). Our pie chart compared the gene function term of overlapped genes against whole gene of human as the background

We found 642 Ensembl genes overlapped with predicted HGT regions and the types of genes were identified according to the Ensemble gene annotation. The most prevalent type of genes was “protein coding”, which covers 39.1% of HGT regions, followed by “lincRNA” (6.6%) and “antisense” (2.9%). The non-gene region covers 46.1% of the HGT regions. Background data (the whole human genome) showed that “protein coding” genes cover 40.7% of human genome, followed by “lincRNA” (7.0%), and “antisense” (3.7%). The non-gene regions cover 45.7% of human genome (Table 2, Fig. 3a). Therefore, the gene type composition of overlapped genes is not significantly different with that of the background.

**Table 2:** The types of genes overlapping HGT regions

Gene type	Background	Overlapped gene
Non gene region	45.7%	46.1%
Protein coding	40.7%	39.1%
LincRNA	7.0%	6.6%
Other	3.0%	5.3%
Antisense	3.7%	2.9%

By using the DAVID to calculate the enrichment of overlapped genes, we discovered 435 genes that had a function annotation in the Gene Ontology (GO) database and categorized these into functional groups and analyzed their enrichment in certain functions. With the modified Fisher Exact test (*p*-value < 0.05), we discovered one term of gene functions are significantly enriched. This term is metal ion binding (120 genes) (Table 3). We also discovered that the proportion of cell part genes is greater than that of the background (Fig. 3c, d). Overall, the most significant gene function impacted by HGT regions is ion binding.

**Table 3:** Gene enrichment analysis of genes overlapping HGT regions by DAVID

Category	Term	gene	*P* Value	Benjamini
GOTERM_MF_FAT	GO:0043167 ~ ion binding	123	1.87E-04	9.12E-02
GOTERM_MF_FAT	GO:0043169 ~ cation binding	121	2.42E-04	5.99E-02
GOTERM_MF_FAT	GO:0046872 ~ metal ion binding	120	2.51E-04	4.19E-02

### Chromatin states of predicted HGT regions

**Fig. 4:**
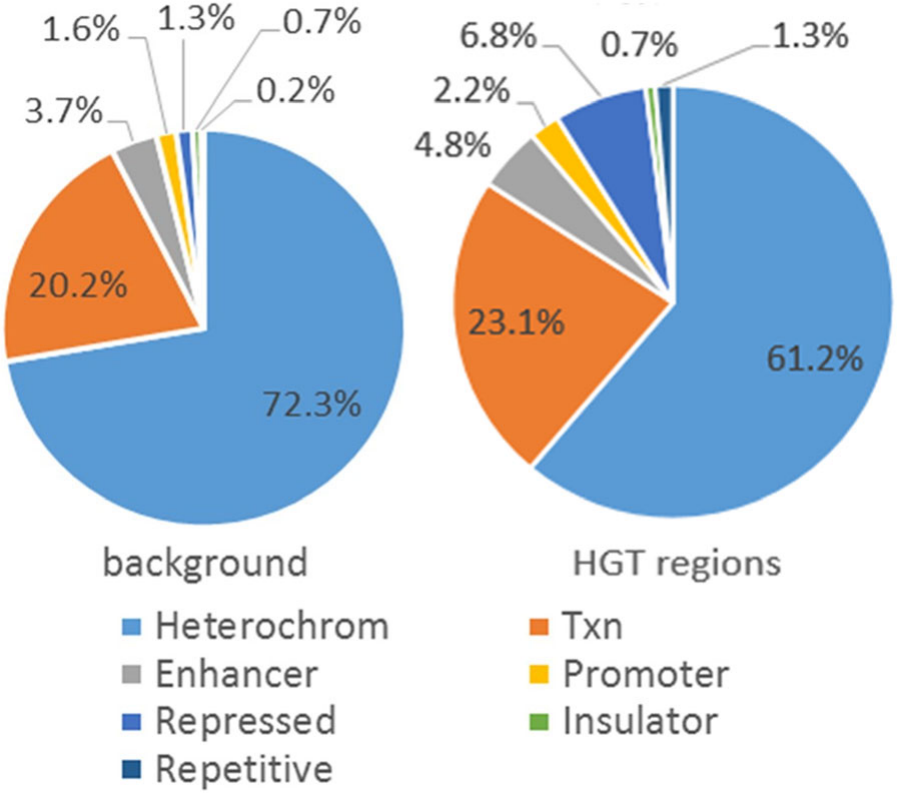
Chromatin states of HGT regions. The chromatin states of predicted HGT sequences included heterochromatin (Heterochrom), transcribed region (Txn), Enhancer, Promoter, Repressed, Insulator and Repetitive. The chromatin state proportions of HGT regions were shown with the whole human genome as the background

Comparing the Chromatin States of predicted HGT regions and human genome, we found that, of the HGT regions, 61% were heterochromatin, 23% were transcription, and 6.8% were repressed. However, in the human genome, 72% were heterochromatin, 20% were transcription, and 1.6% were repressed. (Table 4, Fig. 4). It indicated that HGT regions are more active than the average level of transcription and may play an important functional role in the human genome.

**Table 4:** Chromatin states of predicted HGT regions

Chromatin States	Background	HGT regions
Heterochrom	72.3%	61.2%
Txn	20.2%	23.1%
Enhancer	3.7%	4.8%
Promoter	1.6%	2.2%
Repressed	1.3%	6.8%
Insulator	0.7%	0.7%
Repetitive	0.2%	1.3%

### Repetitive elements in predicted HGT regions

**Fig. 5:**
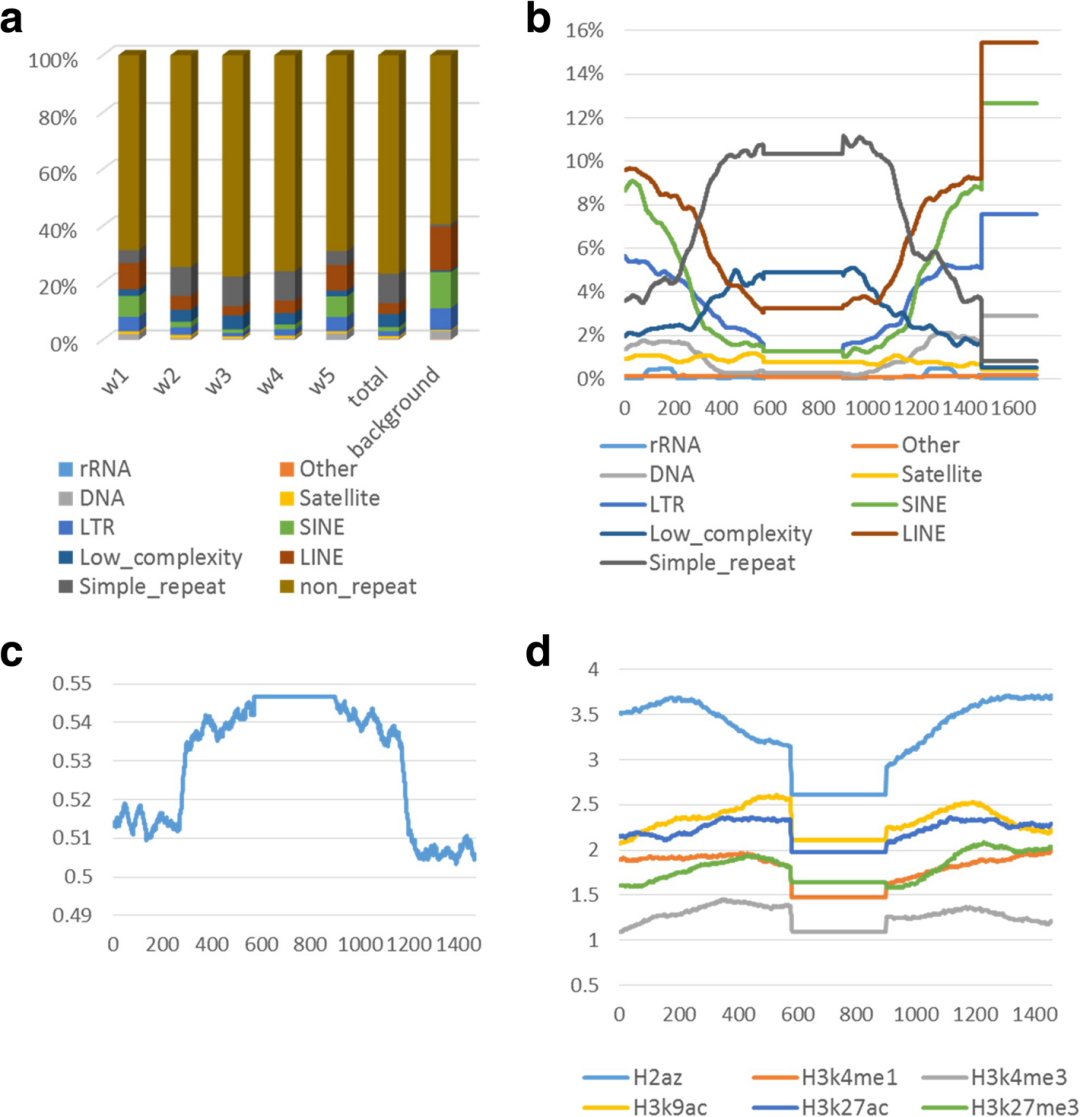
**a** The distributions of repeats of HGT regions. The proportions of different types of repeats, including SINEs, LINEs, LTRs, DNA repeats, simple repeats, low complexity, etc. in predicted HGT regions were shown. Each HGT sequences and franking regions were divided into five regions: window 1 (w1, 300 bps upstream of the sequences), window 2 (w2, the beginning 300 bps of the HGT regions), window 3 (w3, the middle regions of the HGT regions), windows 4 (w4, the ending 300 bps of the sequences), and windows 5 (w5, 300 bps downstream of the sequences). Besides, the distribution of repeat types for HGT regions and the whole human genome. **b** The Distribution of repeats within the HGT regions. We calculated the average proportions of different types of repeats in the five regions of our sequences and drew the average proportions curve of different types of repeats within the HGT regions and 1 kbps upstream and downstream of HGT regions. We can divide the X axis into six intervals: the interval from 0 to 300 bps means the 1 kbps upstream of HGT regions, the interval from 300 bps to 600 bps means the beginning 300 bps of HGT regions, the interval from 600 bps to 900 bps means the middle regions of HGT regions, the interval from 900 bps to 1200 bps means the ending 300 bps of HGT regions, the interval from 1200 bps to 1500 bps means the 300 bps downstream of HGT regions, the interval from 1500 bps to 1700 bps means the whole human genome. **c** and **d** GC contents and histone modification within the HGT regions. We calculated the GC content and drew GC content and histone modification curve for our sequences with a similar approach as the above section we did for repeats in Fig.5c, d. Our histone marks include H3K4Me1, H2A.Z, H3K4Me3, H3K9Ac, H3K27Ac, H3K27Me3, etc

Using the RepeatMasker track, we calculated the percentage of HGT regions that were known repeats, as well as the proportion of each type of repeats (simple repeat, SINE, LINE, etc.). The percentage of repeats (average: 24%) in HGT regions was lower than that of the background region (average: 41%). The most prevalent type of repeats is “simple repeat”, occupying about 10% of the whole HGT regions, followed by “Low complexity repeat” at 4.5%. Background data shows, in human genome, the most prevalent types of repeats are LINE and SINE repeats, with minimal amount of “simple repeats” (Fig. 5a).

Compared to the flanking regions, the predicted HGT regions contains higher percentages of “simple repeat” and “Low complexity repeat”. But for the other types of repeats, such as “LTR”, “SINE” and “LINE” repeats, the percentage in HGT regions is lower than the upstream and downstream regions (Fig. 5b).

### GC contents of predicted HGT regions

We calculated the average percentage of CG content of predicted HGT regions and their 300 bps upstream and downstream regions. The average GC percentage of HGT regions is higher than the upstream and downstream regions (Fig. 5c).

### Histone modification analysis of predicted HGT regions

We calculated the average percentage of regulatory related histone modification signals in predicted HGT regions and their 300 bp upstream and downstream regions. Results showed no significant feature of histone modification in HGT regions (Fig. 5d).

### Phylogenetic tree analysis

Our pipeline can find the HGT regions which have greater conservation with non-mammalian vertebrates than with most mammals. It is also important to note that we allow less than eight mammals containing homologous sequences with the human genome. Indeed, for most HGT regions we have found, some mammals do have homologous sequences with hg19 and most of them are the primates. This phenomenon indicated horizontal gene transfer happened between the ancestor of primates and the non-mammalian vertebrates.

In order to further understand the HGT mechanism, we extracted homologous sequence of the predicted horizontal gene transfer sequences in mammals and non-mammalian species, constructed sequence phylogenetic tree and compared it with species phylogenetic tree. Comparison of two phylogenetic trees showed that, in most cases, two phylogenetic trees were not consistent. Then we compared the tree constructed from predicted HGT regions to the tree constructed from species genomes using the program SPRIT that estimated the number of required subtree prune and regrafts (SPR) to transform one tree into another. It was apparent from the SPRIT output that for most HGT regions, at least three or four SPRs are required to explain the homologous sequences' phylogenetic tree topology. Each SPR may correspond to at least one HGT event, therefore we conclude that at least three or four interspecies HT events have occurred during the evolutionary history of vertebrates. Besides, when we verify the possibility of these HGT events, we found that there were at least one HGT event between the ancestor of all or most primates and the non-mammalian.

**Fig. 6:**
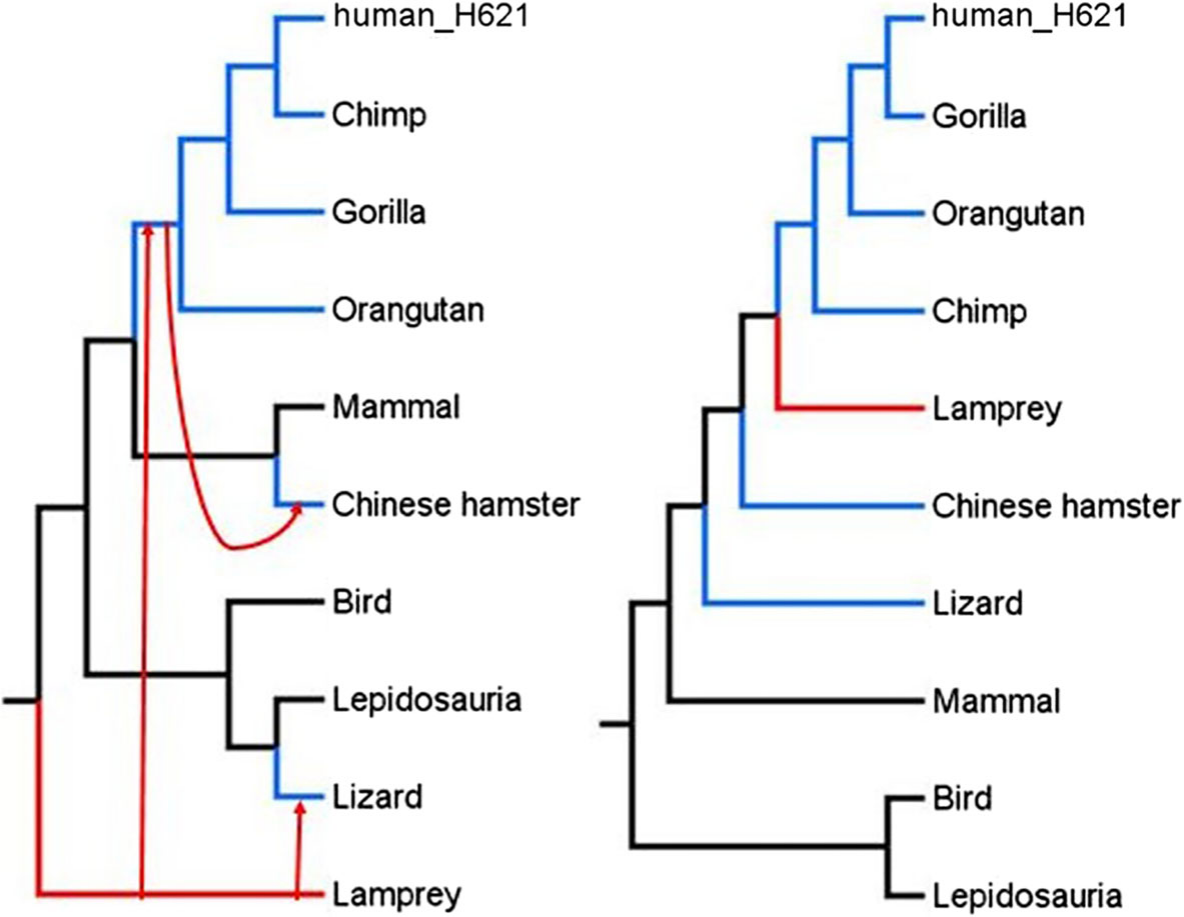
One example of a potential HGT. We aligned the homology sequences of one HGT fragments (range = chr11:71072901–71074379) and constructed the phylogenetic tree. We compared the phylogenetic tree we constructed using homology sequences with the species phylogenetic tree and highlighted the difference between two phylogenetic trees. The red edges represent the origin species of this HGT fragments. The *blue* edges represent the target species to which this HT sequence transferred. The *red* arrows show the horizontal transfer process between two species

With the similarity threshold (60%) and the length coverage (0) between mammalian vertebrates and hg19, we have extracted 22 fragments with lengths more than 1000 bps. These sequences are most likely to be HGT regions. We discovered that one of these fragments, range = chr11:71072901–71074379, has homologues sequence in four primates (Human, Chimp, Gorilla, and Orangutan), one mammal (Chinese hamster), one *Lepidosauria* (Lizard), and one fish (Lamprey). When we have searched the Nucleotide collection database to find the homologous sequences of our HGT regions in other species, we found that several fishes (such as *Cyprinus carpio*, *Lethenteron camtschaticum* etc.) have the homologous sequences, which suggests that fish might be the origin of this HGT region. Comparing the phylogenetic trees built by HGT fragments and species genomes, we discovered at least three predicted HGT events were required to explain the inconsistency. We thought there might be some HGT events happening between fish and Primates, between fish and Lizard or between Primates and Chinese hamster (Fig. 6). Therefore, we concluded that three interspecies HGT events might have occurred during the evolutionary history of this HGT fragment.

### The homologous sequences in media species

We were not able to remove the interpolation of gene lost. However, more evidence can be found to support HGT for these regions we identified. When we searched the Nucleotide collection database to find the homologous sequences of the predicted HGT fragments, we were able to find them in some species, such as parasite, bacteria or fungi, which might be the media species for the HGT progress. Results showed that most of our predicted HGT fragments have homologues sequences in human, fish, bird, poultry and livestock. Among the 22 most reliable predicted HGT regions, 6 have homologues sequences in at least one trematode parasite of human (Table 5), such as *Echinostoma* and *Schistosoma*. Both *Echinostoma* and *Schistosoma* can infect human and other mammals. Some species of Schistosoma can also infect birds and crocodiles. Other trematodes, which though have no homologous sequence of the predicted HGT fragments, can also infect the fishes [29]. Some of our predicted HGT fragments can be find in primates and some species of fishes and birds. From our Phylogenetic tree analysis, we predicted there might be several HGT events happening among fish, bird, primate and a few mammals, which lead us to think that the fishes and birds might be the origin of the HGT progress and trematode parasites or their common ancestor might be the media species for the HGT procedures.

**Table 5:** The homologous sequences of predicted HGT regions in parasites of human

Horizontal transfer sequences	Parasites
chr2:2008034-2009051	*Echinostoma caproni*, *Trichomonas vaginalis*, *Brugia malayi*, *Nippostrongylus brasiliensis*
chr3:175937-177319	*Lottia gigantea*, *Plasmodium yoelii*
chr3:195825850-195827202	*Toxocara canis genome*, *Eimeria maxima*
chr6:141002661-141004363	*Leishmania infantum*, *Neospora caninum*, *Protopolystoma xenopodis*, *Leishmania mexicana*, *Echinostoma caproni*, *Neospora caninum*, *Toxocara canis*, *Ascaris lumbricoides*
chr11:71072902-71074379	*Protopolystoma xenopodis*, *Enterobius vermicularis*, *Nippostrongylus brasiliensis*, *Echinostoma caproni*, *Helicobacter pylori*, *Leishmania infantum*, *Trichobilharzia regenti*, *Taenia asiatica*
chr13:52348752-52349979	*Parascaris equorum*

## Discussions

### The reliability of predicted HGT regions predicted in the human genome

In our pipeline, we can also change the similarity threshold (40%, 50, 60%) between vertebrates and hg19 and the length coverage (40%, 20%, 0) between mammalian vertebrates and hg19 to calculate the number of sequences and the total bases we extracted for HGT regions. With different threshold values, we have discovered different number of sequences. We have discovered 22 HGT regions when we set the similarity threshold to 60% and length coverage to 0. These 22 sequences are more likely to be the result of HGT events. We verified the sequences based on the existence of homologous sequence in several groups of vertebrates using the University of California Santa Cruz (UCSC) Comparative Genomics (Primate Chain/Net, Placental Chain/Net, Vertebrate Chain/Net), finding 19 sequences have an abnormal conservation. When we lowered the similarity threshold to 40% and raised the length coverage to 40%, we have discovered 1,467 predicted HGT regions. In order to verify the result of 1,467 sequences, we sampled 50 of them to check their conservation levels manually in mammals and non-mammalian species using the (UCSC) Comparative Genomics track (Primate Chain/Net, Placental Chain/Net, Vertebrate Chain/Net). We found 44 of the 50 regions (88%) have an abnormal conservation. Therefore, there might be hundreds or even more than a thousand predicted HGT regions in the human genome.

### Comparison against invariant regions among many human genomes

We also analyzed the HGT potential of five invariant regions among all human genomes. None of our HGT regions overlapped with these five sequences, whose corresponding track displays from the UCSC Genome Browser can be found in Table 6. However, using the UCSC Genome Browser, we discovered that we may have missed the 1st sequence because of the presence of too many mammals which were also conserved. If we allowed a few more (than 8) mammals to be existed in our pipeline, we might find part of this invariant region in our results. We may have missed the 2nd and 3rd sequences because the length of the subsequence which aligns the non-mammal with human is shorter than our threshold, 1000 bps.

**Table 6:** The human invariant regions with probably HGT phenomenon

No.	Chromosome	Locations
1	chr2	110590705-110597739
2	chr3	197937579-197944751
3	chr7	74984248-74988311
4	chr7	74930339-74934151
5	chrX	74174167-74181002

### Comparison with other existing HGT regions

There were 145 horizontal transfer genes found in Primates in a previous study [10]. We compared our result with these 145 genes and found that very few (5) genes were in both results. Comparing our pipeline with this previous study, we found that both pipelines extracted those human genome regions that show greater conservation with distant species than with the close species. However, in previous articles, the human genes having greater conservation with non-metazoan than metazoan were identified while our pipeline looked for human genome sequences having greater conservation with non-mammals than mammals. In another words, we looked for HGT events happened more recently than those found in the previous studies. Therefore, very few genes were found in both two results.

### Horizontal gene transfer in Eukaryotes

According to our previous knowledge of biology, horizontal gene transfer in prokaryotes is a common phenomenon, and the underlying mechanism has been extensively studied. In recent years, the studies of horizontal gene transfer in Eukaryotes has gradually increased, though these findings still remain controversy. At present, these previous studies still have many shortcomings, such as lack of evidences to prove these sequences are indeed derived from the horizontal gene transfer, few studies of underlying mechanism, incapability of finding common features of these HGT regions, and so on.

In this study, we not only identified 1,467 HGT regions in the human genome, but also analyzed the phenomenon of horizontal gene transfer in Eukaryotes. We analyzed the conservation of HGT regions in different Eukaryotic genomes to determine whether these sequences are derived from the horizontal gene transfer or not. The analysis of chromosomal locations of these sequences and repeat contents lead us to find some possible features of these HGT regions. We discovered the genes that had any overlap with our extracted sequences and we found most significant gene functions impact by HGT, by using gene function enrichment analysis. Comparing the phylogenetic tree built by homologous sequences with the species phylogenetic tree, we can predict the process of horizontal gene transfer. Besides, we also discovered the homologous sequences in the common parasite of humans and other eukaryotes. It has been reported that horizontal gene transfer in Eukaryotes need some media species to spread the genomic fragments. And these media species might be intracellular parasitic bacterium (*Wolbachia*) or some endoparasites (*schistosomiasis*, *Trypanosoma*) [30]. One study of Schistosoma genome found that *Schistosomiasis* share more orthologues with the vertebrates than they do with the *ecdysozoans* [31]. So the interaction between the host genome and the parasite genome might be a possible step in HGT. We discovered the homologous sequences in the common parasites (*Schistosomiasis*) of humans and other Eukaryotes, which provided the evidence to prove horizontal gene transfer process we predicted is true.

In general, the majority of the HGT regions discovered by our pipeline is credible. The pipeline we built to analyze horizontal gene transfer in Eukaryotes is also reasonable. According to our previous discussion, we believe that horizontal gene transfer is more common than we expected in the human genome. This study will provide a reference for future study of the horizontal gene transfer in Eukaryotes.

## Conclusions

Hundreds of human genes were found to be more conserved with non-mammals than with most mammals. Few of these genes overlapped with known horizontal transferred genes. This indicated that horizontal gene transfer is more common than we expected in the human genome. This study provided insight into potential mechanisms of HGT in the human genome.

## Abbreviations

HGT: Horizontal Gene Transfer
SPIN: Space invaders
SPR: Distance: subtree prune and regraft distance

## References

[1] Freeman VJ (1951). Studies on the virulence of bacteriophage-infected strains of Corynebacterium diphtheriae. Journal of bacteriology.

[2] Syvanen M (1985). Cross-species gene transfer; implications for a new theory of evolution. Journal of theoretical biology.

[3] Jain R, Rivera MC, Lake JA (1999). Horizontal gene transfer among genomes: the complexity hypothesis. Proceedings of the National Academy of Sciences of the United States of America.

[4] Barlow M (2009). What antimicrobial resistance has taught us about horizontal gene transfer. Methods in molecular biology.

[5] Gyles C, Boerlin P (2014). Horizontally transferred genetic elements and their role in pathogenesis of bacterial disease. Veterinary pathology.

[6] Rossi F, Rizzotti L, Felis GE, Torriani S (2014). Horizontal gene transfer among microorganisms in food: current knowledge and future perspectives. Food microbiology.

[7] Chan CX, Bhattacharya D, Reyes-Prieto A (2012). Endosymbiotic and horizontal gene transfer in microbial eukaryotes: Impacts on cell evolution and the tree of life. Mobile genetic elements.

[8] Keeling PJ (2009). Role of horizontal gene transfer in the evolution of photosynthetic eukaryotes and their plastids. Methods in molecular biology.

[9] Pace JK, Gilbert C, Clark MS, Feschotte C (2008). Repeated horizontal transfer of a DNA transposon in mammals and other tetrapods. Proceedings of the National Academy of Sciences of the United States of America.

[10] Crisp A, Boschetti C, Perry M, Tunnacliffe A, Micklem G (2015). Expression of multiple horizontally acquired genes is a hallmark of both vertebrate and invertebrate genomes. Genome biology.

[11] Gilbert C, Cordaux R (2013). Horizontal transfer and evolution of prokaryote transposable elements in eukaryotes. Genome biology and evolution.

[12] El Baidouri M, Carpentier MC, Cooke R, Gao D, Lasserre E, Llauro C, Mirouze M, Picault N, Jackson SA, Panaud O (2014). Widespread and frequent horizontal transfers of transposable elements in plants. Genome research.

[13] Walsh AM, Kortschak RD, Gardner MG, Bertozzi T, Adelson DL (2013). Widespread horizontal transfer of retrotransposons. Proceedings of the National Academy of Sciences of the United States of America.

[14] Lander ES, Linton LM, Birren B, Nusbaum C, Zody MC, Baldwin J, Devon K, Dewar K, Doyle M, FitzHugh W (2001). Initial sequencing and analysis of the human genome. Nature.

[15] Ernst J, Kellis M (2010). Discovery and characterization of chromatin states for systematic annotation of the human genome. Nature biotechnology.

[16] Ernst J, Kheradpour P, Mikkelsen TS, Shoresh N, Ward LD, Epstein CB, Zhang X, Wang L, Issner R, Coyne M (2011). Mapping and analysis of chromatin state dynamics in nine human cell types. Nature.

[17] Kent WJ, Baertsch R, Hinrichs A, Miller W, Haussler D (2003). Evolution’s cauldron: duplication, deletion, and rearrangement in the mouse and human genomes. Proceedings of the National Academy of Sciences of the United States of America.

[18] Karolchik D, Barber GP, Casper J, Clawson H, Cline MS, Diekhans M, Dreszer TR, Fujita PA, Guruvadoo L, Haeussler M (2014). The UCSC Genome Browser database: 2014 update. Nucleic acids research.

[19] Chiaromonte F, Yap VB, Miller W. Scoring pairwise genomic sequence alignments. Pacific Symposium on Biocomputing Pacific Symposium on Biocomputing. 2002;7:115–26. 10.1142/9789812799623_0012 11928468

[20] Schwartz S, Kent WJ, Smit A, Zhang Z, Baertsch R, Hardison RC, Haussler D, Miller W (2003). Human-mouse alignments with BLASTZ. Genome research.

[21] Flicek P, Amode MR, Barrell D, Beal K, Billis K, Brent S, Carvalho-Silva D, Clapham P, Coates G, Fitzgerald S (2014). Ensembl 2014. Nucleic acids research.

[22] da Huang W, Sherman BT, Lempicki RA (2009). Systematic and integrative analysis of large gene lists using DAVID bioinformatics resources. Nature protocols.

[23] da Huang W, Sherman BT, Lempicki RA (2009). Bioinformatics enrichment tools: paths toward the comprehensive functional analysis of large gene lists. Nucleic acids research.

[24] Barski A, Cuddapah S, Cui K, Roh TY, Schones DE, Wang Z, Wei G, Chepelev I, Zhao K (2007). High-resolution profiling of histone methylations in the human genome. Cell.

[25] Blanchette M, Kent WJ, Riemer C, Elnitski L, Smit AF, Roskin KM, Baertsch R, Rosenbloom K, Clawson H, Green ED (2004). Aligning multiple genomic sequences with the threaded blockset aligner. Genome research.

[26] Murphy WJ, Eizirik E, O’Brien SJ, Madsen O, Scally M, Douady CJ, Teeling E, Ryder OA, Stanhope MJ, de Jong WW (2001). Resolution of the early placental mammal radiation using Bayesian phylogenetics. Science.

[27] Tamura K, Stecher G, Peterson D, Filipski A, Kumar S (2013). MEGA6: Molecular Evolutionary Genetics Analysis version 6.0. Molecular biology and evolution.

[28] Hill T, Nordstrom KJ, Thollesson M, Safstrom TM, Vernersson AK, Fredriksson R, Schioth HB (2010). SPRIT: Identifying horizontal gene transfer in rooted phylogenetic trees. BMC evolutionary biology.

[29] Brant SV, Loker ES (2005). Can specialized pathogens colonize distantly related hosts? Schistosome evolution as a case study. PLoS pathogens.

[30] Schaack S, Gilbert C, Feschotte C (2010). Promiscuous DNA: horizontal transfer of transposable elements and why it matters for eukaryotic evolution. Trends in ecology & evolution.

[31] Schistosoma japonicum Genome S, Functional Analysis C: The Schistosoma japonicum genome reveals features of host-parasite interplay. Nature 2009, 460(7253):345–351. 10.1038/nature08140 PMC3747554 19606140

